# Inter-implant distance correlated to different preparation protocol on cortical bone: an animal study

**DOI:** 10.1186/s12903-025-07028-5

**Published:** 2025-10-02

**Authors:** Marco Toia, Michele Stocchero, Yohei Jinno, Manjula Herath, Silvia Galli, Evaggelia Papia, Marianne Ahmad, Jonas P. Becktor

**Affiliations:** 1 Department of Oral and Maxillofacial Surgery and Oral Medicine, Faculty of Odontology, Malmö University, Malmö, Sweden; 2 Section of Implant and Rehabilitative Dentistry, Division of Oral Rehabilitation, Faculty of Dental Science, Kyushu University, Fukuoka, Japan; 3 Department of Materials Science and Technology, Faculty of Odontology, Malmö University, Malmö, Sweden

**Keywords:** Inter-implant distance, Undersized preparation, Bone-to-implant contact, Bone Mineral density

## Abstract

**Background:**

Inter-implant distance (IID) plays a crucial role in maintaining peri-implant bone stability and osseointegration. Narrower IIDs (< 3 mm) have been associated with increased bone loss, but the threshold varies with implant connection type. The undersized preparation protocol, which induces static strain in the bone, enhances primary stability but may lead to microstructural damage, remodelling, or necrosis. The effect of this technique on osseointegration in adjacent implants with varying IIDs is not well documented. This study investigates the impact of undersized and non-undersized preparation protocols on osseointegration at different IIDs.

**Methods:**

The study utilized eight sheep, with 64 implants placed in the mandibles following two surgical protocols: undersized preparation (UP) and non-undersized preparation (NUP). Implants were positioned at two IIDs (2 mm and 4 mm). Biomechanical, histomorphometric, and micromorphometric analyses were performed five weeks post-surgery. Bone-to-implant contact (BIC), bone volume fraction (BVTV), and bone mineral density (BMD) were measured in the regions of interest (inner and outer portions relative to the IID). A linear mixed model approach was applied to analyse the data with statistical significance set at *p* < 0.05.

**Results:**

The analyses showed no statistically significant differences between the surgical protocols or IIDs for the evaluated parameters (*p* >0.05). Nevertheless, trends were observed, with higher BIC and increased bone remodelling in the inner regions at 2 mm IID, particularly when using the undersized preparation protocol. Additionally, BVTV values were higher in the inner portions at 4 mm IID, suggesting reduced bone remodelling compared to 2 mm IID. These results indicate that while mechanical stress influenced trends in bone response, the overall resilience of peri-implant bone healing was evident.

**Conclusions:**

No significant differences in osseointegration were observed between surgical protocols or IIDs. However, the trend of increased remodelling and higher BIC at 2 mm IID highlights the mechanical impact of undersized preparation in close implant spacing. These findings emphasize the complexity of peri-implant bone response to mechanical forces, necessitating further clinical studies to validate these results in human models.

**Supplementary Information:**

The online version contains supplementary material available at 10.1186/s12903-025-07028-5.

## Introduction

In multiple dental implant rehabilitations, a minimum distance between adjacent implants is considered critical to achieve a stable bone crest level and aesthetic outcome [1]. However, some anatomical conditions require implants to be placed in close proximity, for example in a narrow ridge, which may restrain the peri-implant bone compartment and thus prospect bone loss and soft tissue retractions [2, 3].

Tarnow at al. [4] reported that the bone crest was more apically located at sites where the inter-implant distance (IID) was less than 3 mm. In another retrospective study [5] where the IID varied between 0.5 mm and 6.0 mm with a mean range of 3.2 mm, it was reported more bone loss when the IID was less than 3 mm. According to Chang et al., 3 mm was considered to be the minimum IID when external hexagon implants were used [6].

However, when conical connection implants were used, 1.8 mm was considered as the IID threshold for bone crest maintenance [7, 8].

In dental implantology, achieving optimal implant primary stability is paramount, and one prevalent protocol employed is undersized preparation. This method entails the preparation of the implant site with a diameter markedly less than that of the implant itself [9].

Such a procedure necessitates the implant to mechanically engage with the bone by cutting and compressing the bone walls during insertion, thereby inducing static strain within the peri-implant bone matrix. This mechanical interlock, or press-fit condition, is critical for the stabilization of the implant in the initial osseointegration phase [10].

However, it is crucial to note that excessive mechanical expansion, experiencing non-elastic strain due to implant placement, can lead to plastic deformation of the peri-implant bone, potentially initiating microfractures [11]. The resultant biomechanical stress may precipitate local ischemic conditions, cellular trauma, and thermal injury to the bone tissue [12, 13]. In severe instances, this can culminate in osteocyte necrosis, particularly when the applied pressure exceeds the tolerance of the cortical bone [14].

The clinical implications of undersized preparation, include potential early implant failure due to pressure-induced necrosis or marginal bone resorption. A previous study in this domain have documented extensive bone remodelling triggered by the undersized preparation protocol, even in the absence of mechanical loading. Initially, this remodelling process is characterized by osteoclastic activity aimed at resorbing the bone subjected to stress and damage, which might transiently diminish the primary stability of the implant [15]. However, depending on the bone quality the effect of the surgical technique could damage the coronal portion of the bone during the healing phase [15]. Higher lateral bone compression does not always enhance mechanical stability and osseointegratiofVolume of interestcrosis and generate microfractures [16].

The undersized preparation protocol is particularly used in case of immediate loading procedure to maximize the implant’s stability [17].

However, the effect of the osteointegration in undersized adjacent implants is not well documented.

It is hypothesized that implants placed using undersized preparation protocol with limited IID will affect the osseointegration process.

The objective of this animal study is to analyse if different preparation protocols (undersized or non-undersized) could affect the osseointegration process of implants placed with two different IID by means of biomechanics, histomorphometric and micro-morphometric analyses.

## Materials and methods

### Surgical procedures

The study protocol was approved by the animal research committee in Ecole Nationale Vétérinaire d’Alfort (Maisons-Alfort, Val-de-Marne, France) (reference number: E940462-2019), and it is reported according to the ARRIVE (Animal Research Reporting of in VIVO Experiments) guidelines (Berglundh & Stavropoulos, 2012). The sheep used in this study were sourced from a farm that collaborates with the university’s research centre. The farm provided the animals in an established partnership with the centre. In accordance with the approved research protocol, the farm owners gave written informed consent to use the animals in this study. All animal experiments were performed in Ecole Nationale Vétérinaire d’Alfort (Maisons-Alfort, Val-de-Marne, France).

The study was designed as a controlled experimental study with four experimental groups. Sixty-four dental implants (AstraTech EV 3.6 mm × 6 mm, DentsplySirona, Mölndal, Sweden) were installed into the inferior boarder of the mandible body, consisting of approximately 3 mm of cortical bone thickness, in eight female Finnish Dorset sheep (average age: 2 years and 1 month; average body weight ± SD: 52.04 ± 5.12 kg).

All the surgical procedures were performed under general anesthesia with ketamine (6 mg/kg, Imalgene ND by Merial, Merial SAS, Villeurbanne, France) and diazepam (0.5 mg/kg, Valium ND by Roche, Roche, Boulogne-Billancourt, France). After endotracheal intubation, anesthesia was maintained with 2.5% isoflurane (Forane®/Forene®, Drägerverk AG, Lübeck, Germany).

**Fig. 1:**
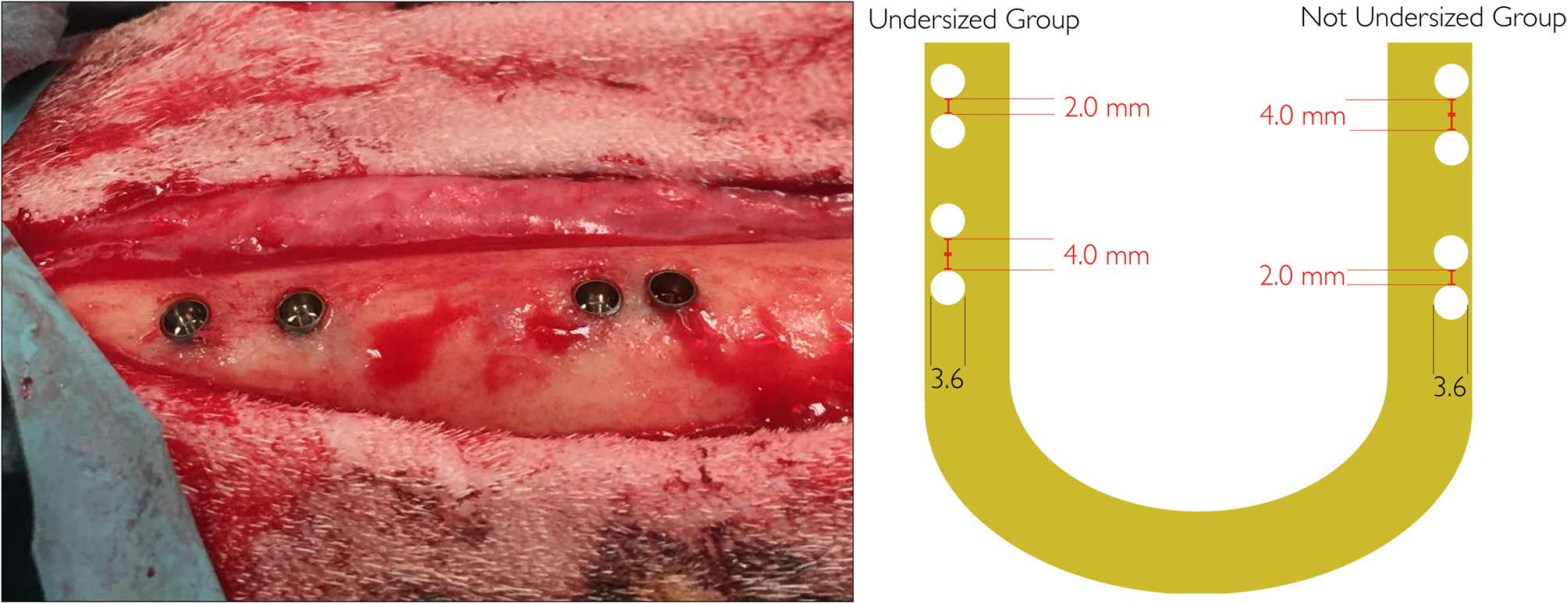
Experimental implant distribution at the inferior border of the mandibular body. Each hemimandible of the sheep was used to accommodate both undersized preparation (UP) and non-undersized preparation (NUP) groups, with implants placed at varying inter-implant distances (IDs). A schematic representation of the implant positions and preparation protocols is shown for clarity on the right side

Preoperatively, surgical areas were shaved, disinfected with iodine solution and draped. After skin incision down to periosteum, both sides of the inferior and lateral boarder of the mandible body (Fig. 1) and ramus were exposed with a periosteal elevator. Implant sites were prepared according to two different preparation protocols and with two different IID, 2 mm and 4 mm by using surgical guides.

Preparation protocols were performed as follows:

- Group NUP, non-undersized preparation: 1.9 mm twist drill was followed by 2.5/3.1 mm step drill. The coronal cortical bone was prepared to 3.6 mm (0 mm undersized preparation) for 2.5 mm length using a full diameter size cortical drill (cortical drill type B, AstraTech EV 3.6 mm 6 mm, DentsplySirona, Mölndal, Sweden).
- Group UP, undersized preparation: 1.9 mm twist drill was followed by 2.5/3.1 mm step drill. The coronal cortical bone was prepared to 3.3 mm (0.3 mm undersized preparation) for 2.5 mm length using a full diameter size cortical drill (cortical drill type A, AstraTech EV 3.6 mm 6 mm, DentsplySirona, Mölndal, Sweden).

After computer-generated randomization, each side of the mandible received two implants from Group NUP or Group UP with 2 mm and 4 mm IID respectively.

All preparation protocols followed the manufacturer recommended drilling speed (1,500 rpm) under sterile saline irrigation to avoid overheating. All implants were installed at the level of the crestal bone (SA-310 W&H Elcomed implant unit, W&H, Burmoos, Austria). In case the insertion torque (ITQ) exceeded 80 Ncm (limit of the implant unit), implants were installed manually by manual torque wrench. ITQ values were recorded and the flap was closed and sutured in two layers with a resorbable suture (Vicryl, Ethicon, INC., Somerville, NJ, USA) for the subcutaneous layer and with a non-resorbable suture (Ethilon, Ethicon, INC., Somerville, NJ, USA) for skin. Benzylpenicillin 114 mg, dihydrostreptomycin 164 mg, and procaine 13 mg (Peni Dhs Coophvet, M.C. Sante Animale, Mohammedia 28810, Morocco) were administered for five days postoperatively, and meloxicam (0.4 mg/kg, Metacam^®^, Boehringer Ingelheim Vetmedica, Ingelheim, Germany) was administered for three postoperative days. Non-resorbable sutures were removed after ten days of healing.

### Collection of samples

Five weeks after surgery, all sheep were euthanized with intravenous overdose injection of a combination of 4 mg embutramide, 538.4 mg mebezobium, and 87.8 mg tetracaine (T61, Intervet International, Unterschleißheim, Germany), and mandibular bone blocks containing the implants were retrieved. Each implant pair (2 mm and 4 mm IID) was isolated in one sample block and cleaned of soft tissue remnants, followed by a preservation procedure of formaldehyde fixation, alcohol dehydration and resin embedding, prior to micro-CT acquisition and later histomorphometric analysis.

### Micro-CT acquisition

The resin embedded sample blocks were scanned with a high-resolution micro-CT (U-CTXUHR, MILabs). The samples were positioned in the sample holder with the implant long axis parallel to the X-ray source to reduce artefact formation in the peri-implant bone volume. Scanning was performed at 65 kV and 130µA, using aluminium filters (a 150 µm filter added to a 100 µm fixed filter) and the exposure time set to 90ms. The 3D image reconstruction was performed with MILabs Reconstruction 11.02 software (MILabs) at a nominal voxel size of 10 µm.

**Fig. 2:**
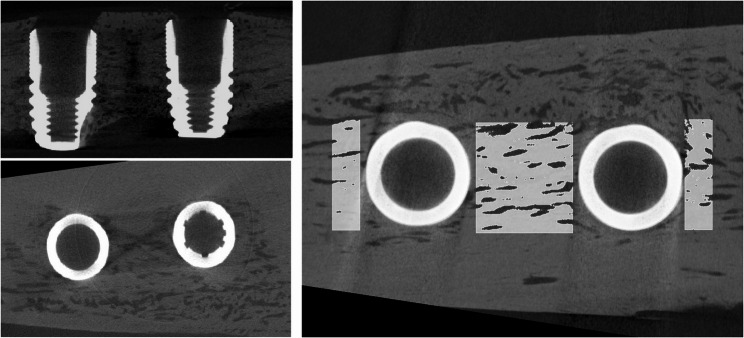
Sections of sample blocks containing implants at 2 mm IID (inter-implant distance).The right side of the image indicates the regions of interest (ROI) designated as inner and outer

The reconstructed volumes were exported in nii-format to 3D Slicer open source software (https://www.slicer.org/), for further bone volume and bone mineral density assessment. (Fig. 2)

### Bone volume (BV/TV) analysis

Before segmentation, threshold levels for bone and implant were determined, based on visual inspection of the volume. Distinctive threshold intervals were defined for implant and bone tissue, consistently applied for all samples. Volumes of interest (VOIs) were defined covering the IID and the counter proximal portions to the implants, creating three VOIs for each sample; one inter-implant volume (inner volume) and two counter-proximal volumes (outer volume).

The entities to calculate bone volume fraction (BV/TV) for each VOI was extracted through the scalar volume statistics module.

### Bone mineral density (BMD) analysis

The U-CTXUHR system was pre-calibrated to attenuation units (Hounsfield units (HU)). A calibration curve based on linear regression was further set to convert HUs into BMD units (g/cm3) using commercially available hydroxyapatite (HA) phantoms (Bruker-MicroCT) as described by Sasser et al. The scalar volume statistics module was used to derive the mean scalar values to each VOI.

### Histology and histomorphometric analysis

The two paired implants with surrounding bone tissue were fixed in 4% buffered formalin for 14 days. The samples were dehydrated in a graded series of alcohol and embedded in light-curing acrylic resin (Technovit 7200 VLC, Heraeus Kulzer, Wehrheim, Germany). Resin-infiltration was initiated with a mixture of ethanol and Technovit 7200 VLC, followed by infiltration with pure Technovit 7200 VLC. The polymerization was done in a photopolymerization unit (EXAKT Apparatebau, Norderstedt, Germany) with exposure to daylight for one hour and to ultraviolet for 2 h.

After light polymerization, specimens were cut parallel to the longitudinal implant axis along the center of two implants, using a high speed, water cooled saw with diamond coating (EXAKT 300CP, EXAKT Apparatebau, Norderstedt, Germany). The cut surface was glued on to plastic slide with Technovit 7210 VLC (Heraeus Kulzer, Wehrheim, Germany) and polymerized with a lighting machine (EXAKT-precision adhesive system, EXAKT Apparatebau, Norderstedt, Germany). Subsequently, the glued block was cut into a 150 µm thick slice using a diamond saw cutting machine (EXAKT 300CP, EXAKT Apparatebau, Norderstedt, Germany) and polished to a 40 µm thickness by a micro grinding system (EXAKT 400CS, EXAKT Apparatebau, Norderstedt, Germany). The non-decalcified ground sections were stained with toluidine blue and pyronin G, and were photographed using digital slide scanner (NanoZoomer S360, Hamamatsu Photonics, Hamamatsu, Japan).

The histomorphometric evaluation was performed in a standard manner by one blinded and calibrated examiner, using a specific image analysis software (ImageJ version: 2.0.0-rc-69/1.52p, NIH, Bethesda, MD, USA).

**Fig. 3:**
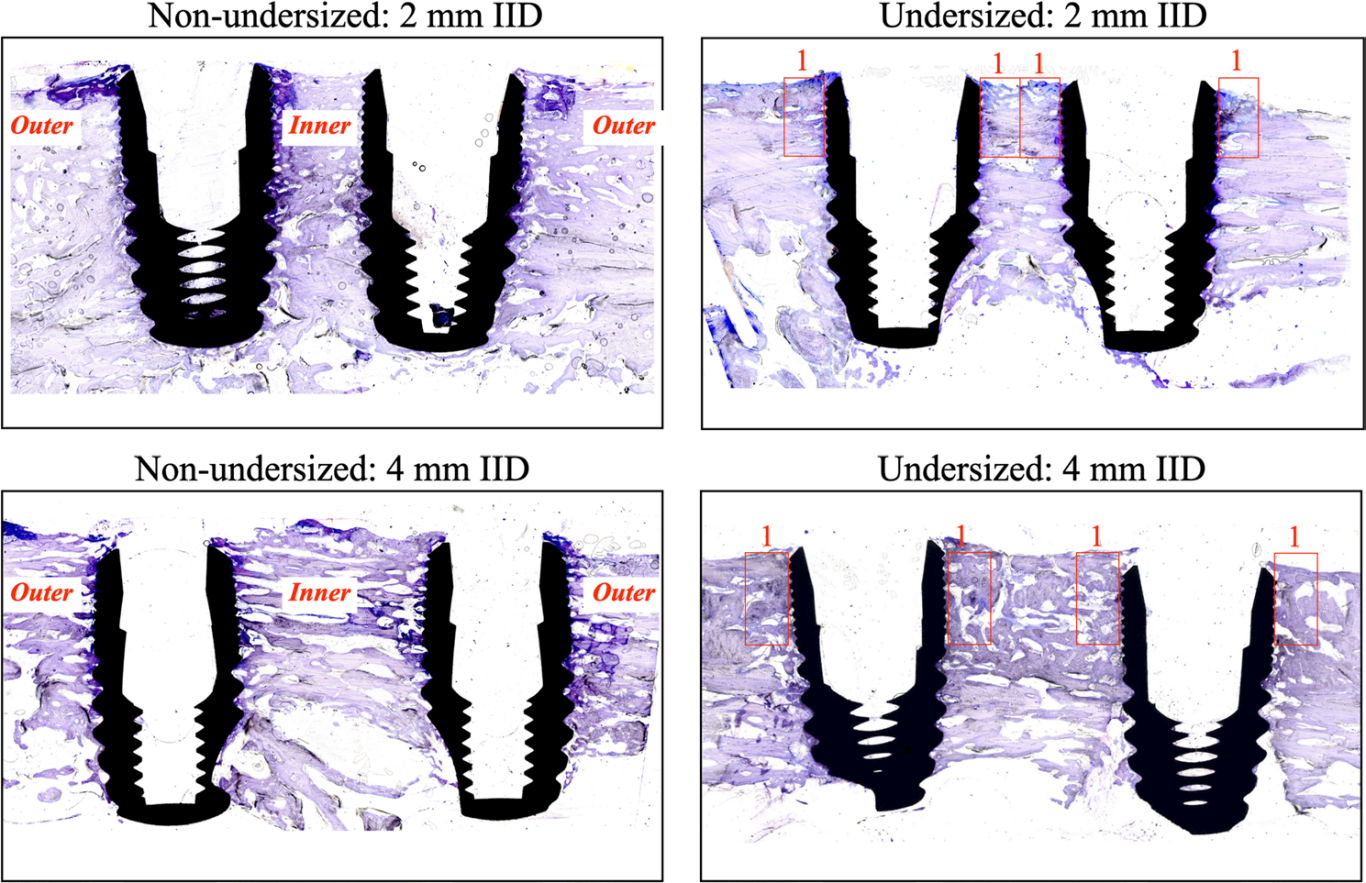
Sections of sample blocks containing implants at 2 mm and 4 mm IID (inter-implant distance) in both non-undersized and undersized groups for the histomorphometric evaluation.The left side of the image indicates the regions of interest (ROI) designated as inner and outer. The right side of the image denotes the 1 mm ROIs

For the sections with implant, to analyse the bone in intimate contact with the implant surface, two histomorphometric parameters were considered in two regions of interests (ROIs) on each side of the implant. The histomorphometric parameters between the two implant sides within the IID were designated as “inner” while the other side of the implant, not involved in the IID as “outer”. Representation of the histologic sections are displayed in Fig. 3. Only the coronal 2.5 mm of the bone was considered.

- Bone-to-implant contact (BIC) (%): The bone tissue in intimate contact with implant surface in ROI (inner and outer).
- Bone area fraction occupancy (BAFO) (%): The percentage of the area inside the implant threads in ROI (inner and outer). The area was calculated at from the implant surface.

The mean value of two measurements for BIC and BAFO for two implants was used for each experimental group in each sheep.

### Statistical analysis

Data management and statistical analysis were performed using Jamovi version 2.3.26 on the dependent variables and following outcomes: BIC_outer; BIC_inner; BAFO_1mm_outer; BAFO_1mm_inner; BVTV_inner; BMD_inner; BVTV_outer; BMD_outer. Given the hierarchical structure of the data with implants nested in sheep, observations could not be assumed to be independent. Accordingly, a mixed linear modelling (MLM) approach was applied to evaluate the differences between groups for the dependent variables listed above. In detail, a 2-level hierarchical structure in which implants represented the units of analysis and sheep represented the upper- class clustering variable. Intercepts were allowed to vary across sheep. The variables for the preparation protocol (UP vs. NUP) and the IID (2 mm vs. 4 mm) were included as predictors, along with their interaction. Mixed modelling analysis were performed using the restricted maximum likelihood estimation (REML) with Kenward-Rogers correction which is the preferable approach when the number of clusters (i.e., sheep) is fewer than 30 [18]. Statistical significance was set at a p-value of 0.05.

## Results

All sheep recovered from the surgical procedures and survived throughout the experimental period. There were no signs of infections or other adverse events in the area of interest, and all the implants were considered as osseointegrated at the end of the experimental observation.

Descriptive statistics for the dependent variables are presented in Table 1 and the parameters estimates for the LMM analyses in Table 2. As it is shown in Table 2, the linear mixed model analyses revealed nonsignificant effects of group (*ps* >0.20) and distance (*ps* >0.06), as well as nonsignificant interaction effects (*ps* >0.13) for all the dependent variables.

**Table 1:** – MLM parameters estimates: effects and (standard error)

Model	BIC_outer	BIC_inner	BAFO_1mm_outer	BAFO_1mm_inner	BVTV_inner	BMD_inner	BVTV_outer	BMD_outer
**Fixed Part**								
Intercept	0.63 (0.04)	0.62 (0.04)	84.71 (1.35)	85.71 (1.77)	71.05 (2.47)	1.04 (0.01)	73.67 (2.19)	1.03 (0.01)
Group	0.02 (0.04)	0.03 (0.04)	2.24 (1.96)	1.29 (1.75)	3.42 (2.62)	−0.04 (0.02)	3.69 (2.65)	−0.01 (0.03)
Distance	0.01 (0.03	−0.04 (0.04)	0.23 (1.91)	1.62 (1.69)	5.06 (2.53)	−0.02 (0.02)	2.39 (2.58)	0.00 (0.03)
Group*Distance	0.00 (0.07)	−0.07 (0.07)	3.88 (3.82)	4.57 (3.38)	−3.29 (5.07)	−0.05 (0.04)	−0.80 (5.16)	−0.06 (0.05)
**Random Part**								
s^2^_e_	0.01	0.01	8.71	23.80	43.89	0.00	30.55	0.00
s^2^_u0_	0.02	0,02	60.68	47.50	57.02	0.00	118.11	0,01
**Model statistics**								
ICC	0.36	0.32	0.13	0.33	0.43	0.25	0.21	0,00
Marginal R^2^	0.00	0.02	0.03	0.03	0.10	0.10	0.03	0.02
Conditional R^2^	0.36	0.34	0.15	0.35	0.49	0.32	0.23	0.02

*Notes.* s^2^_e_ = intercepts level variance; s^2^_u0_ = Residual variance; ICC = intraclass correlation coefficient; Marginal R^2^ = variance explained by fixed effects only; Conditional R^2^ = variance explained by both fixed effects and random intercept

**Table 2:** Mean values of histomorphometric analysis

	Inter-implant distance							
	2 mm				4 mm			
	Non-undersized		Undersized		Non-undersized		Undersized	
	*N*	Average (SD)	*N*	Average (SD)	*N*	Average (SD)	*N*	Average (SD)
BIC outer (%)	19	61,98 (16,48)	16	64,53 (14,18)	19	62,85 (21,41)	16	65,17 (16,45)
BIC inner (%)	20	60,83 (18,05)	15	67,16 (19,68)	20	60,03 (20,25)	16	60,13 (15,94)
BIC TOT (%)	19	61,97 (13,97)	15	65,56 (13,31)	19	62,21 (19,11)	16	62,65 (15,58)
BIC Diff (outer-inner) (%)	19	0,02 (19,91)	15	−3,2 (2,2)	19	1,28 (14,85)	16	5,04 (8,83)
BAFO outer 1 mm (%)	19	84,47 (8,69)	16	84,79 (5,93)	19	82,76 (11,02)	14	87,17 (5,25)
BAFO inner 1 mm (%)	20	85,39 (8,15)	15	84,12 (4,9)	20	84,73 (11,24)	14	89,54 (5,79)
BAFO 1 mm Diff (outer-inner) (%)	19	−0,96 (9,62)	15	0,54 (5,02)	19	−2,65 (10,59)	14	−2,37 (8,33)
BVTV outer (%)	20	70,44 (17,81)	16	74,49 (6,73)	20	73,22 (9,99)	16	76,48 (10,01)
BVTV inner (%)	10	65,99 (10,15)	8	71,76 (9,54)	10	72,69 (9,51)	8	75,18 (11,35)
BVTV Diff (outer-inner) (%)	10	4,07 (16,26)	8	2,85 (7,74)	10	−0,91 (6,72)	8	0-,11 (3,38)
BMD outer	20	1,01 (0,19)	16	1,04 (0,05)	20	1,04 (0,05)	16	1,01 (0,1)
BMD inner	10	1,06 (0,05)	8	1,05 (0,06)	10	1,06 (0,05)	8	1,01 (0,1)
BMD Diff (outer-inner)	10	−0,08 (0,26)	8	−0,02 (0,05)	10	−0,02 (0,02)	8	0 (0,02)

*Notes*. BIC (Bone-to-implant contact); BAFO (Bone area fraction occupancy); BVTV (Bone volume/Total volume); BMD (Bone mineral density)

## Discussion

Maintaining an adequate inter-implant distance (IID) is widely recognized as critical for preserving peri-implant bone and soft tissue. A recent systematic review confirmed that a horizontal IID of at least 3 mm is essential to minimize marginal bone loss and support healthy papillae between adjacent implants [19]. Tarnow et al. [4] and others similarly reported greater crestal bone resorption when implants are placed too close (< 3 mm apart).

The hypothesis of the present study that narrower IID of 2 mm leads to increased bone remodelling and resorption at 5 weeks in comparison with IID of 4 mm, was rejected since there was no statistical significance. Even though, the inner portions at an IID of 2 mm, BVTV and BIC showed no statistical significance, the results revealed a trend of increased bone remodelling and resorption in comparison to the external portion of the implants. The BVTV inner portion indicates a greater bone volume fraction when the IID is 4 mm. This data is in line with a finite element analysis study which reported that 10 mm is the optimal distance between two implants in order to create a better stress distribution in the bone while an IID of 5 mm may increase the stress distribution around the cortical bone [20].

Regarding the BIC in the inner portion, it was observed to be higher when implants are spaced 2 mm apart. This increase of BIC was even more significant when an undersized preparation protocol was used. This may be explained by the effect of bone compression due to the undersized preparation protocol and the narrow distance between the implants. These findings align with the results from other animal studies, which have reported that implants installed with high static strain exhibit increased BIC values [21].

However, the bone, compressed against the inner surface of the two implants, is highly susceptible to undergo plastic deformation at the microstructural level, which in turn increases the probability of bone resorption [15]. It is noteworthy that the excessive static strains introduced during the implant installation process do not necessarily impede the long-term progression of osseointegration. This observation holds true even in the context of the initial intensive bone remodelling and necrotic events, underscoring the complex interplay between mechanical forces and biological healing in dental implantology [22, 23].

The 5-week endpoint used in the present study corresponds to the transitional period between the proliferative and early remodelling phases, as identified in preclinical models [24]. Although the complete A-R-F cycle (Activation–Resorption–Formation) [24] spans approximately 80 days, this early phase is crucial for evaluating the initial biological response to surgical trauma and mechanical strain, which may vary across implant protocols. Our previous findings [15, 21]confirmed that microstructural changes and bone turnover markers are already detectable at this stage in sheep models.

The 0.3 mm undersized preparation was selected based on our prior validated model [15], which demonstrated its efficacy in producing detectable differences in strain-related remodelling without inducing excessive necrosis. This value reflects a clinically common degree of undersizing aimed at enhancing primary stability. A recent in vitro experiment systematically compared undersizing by 0.2 mm, 0.5 mm, and 0.8 mm; interestingly, it found that a small undersize (~ 0.2 mm, ~ 6% of implant diameter) was sufficient to improve primary stability, and larger undersizes did not further increase stability [25].

While comparative evaluation of multiple undersizing levels could offer additional insights, we prioritized methodological control and reproducibility in the context of the same animal model reported in our previous studies [15, 21].

Microdamage resulting from localized static strain may act as a biological trigger for bone turnover, especially in undersized sites [23].

During implant insertion, especially when two implants are in close proximity, their individual strain fields in the bone may overlap and concentrate in the inter-implant septum. Moreover, the combination of a reduced inter-implant space and the high insertion torque from undersized drilling induced a stronger remodeling response in the adjacent bone. Biomechanically, the undersized preparation protocol generates high static strains in the surrounding cortical bone as the implant is inserted with interference fit and can induce micro-cracks and microscopic damage in the bone tissue. These microinjuries trigger a cascade of biological events: osteocyte apoptosis in the damaged area leads to the recruitment of osteoclasts (via signaling such as RANK–RANKL) to remove the compromised bone, followed by osteoblastic formation of new bone (the Activation–Resorption–Formation remodeling sequence [24]). In essence, the bone’s response to microdamage from static load is to remodel itself – resorbing and replacing the damaged tissue and underpreparation can generate micro-fractures and a remodelling response by Basic Multicellular Units (BMUs) [15]. Markers of this process include osteoclast activity markers (e.g. tartrate-resistant acid phosphatase, TRAP, indicating resorption) and subsequent osteoblast activity markers (e.g. osteocalcin for new bone formation). Although our study did not measure these molecular markers directly, the histological findings strongly imply their involvement. These findings align with Slaets et al. [26] who reported that even in the absence of external load, an implant can exert static mechanical strain on surrounding bone, influencing remodelling activity. Although microdamage was not directly quantified, these findings align with prior reports from our group and with biomechanical models of bone adaptation [15, 16, 21].

Toluidine blue staining revealed distinct patterns of maturation. This histological method represents a reliable and widely accepted indicator of ongoing bone remodelling processes: areas of new bone (dark blue) indicate where bone formation has recently occurred, often replacing earlier resorbed bone, whereas areas of light-blue lamellar bone indicate older, pre-existing bone that has not been turned over in the current remodelling cycle. Toluidine blue is not just a general stain but a purposeful tool in our study to assess the state of bone maturation in the context of the healing timeline. It reinforces our findings that, at 5 weeks, much of the bone around the implants is newformed (woven), aligning with the expectation that the full remodelling (replacement with mature bone) is incomplete at this stage.

From a clinical standpoint, it is essential to exercise caution during immediate loading, especially when installing two implants in close proximity with high torque due to undersized preparation protocol. Consequently, the initial BIC which might appear to have a high value might be insufficient already after 5 weeks of healing and thereby challenging to interpret.

A clinical study reported by Degidi et al. [17] involving forty-nine individuals needing replacement of two or more adjacent teeth with implants showed that implants with an IID of less than 2 mm exhibited less bone loss compared to those placed further apart. However, in the referenced study, implants were inserted into post-extraction sites. This specific procedure suggests that the crestal module of the implant may interact with the cortical layer, potentially influencing a minimal segment of the cortical bone, if at all [27]. Such an interaction is likely to induce a standard wound healing process at the post-extraction site [28]. Furthermore, this scenario is characterized by the release of relatively low biomechanical stress at the cortical layer, accompanied by minimal strain, which is a critical factor in the osseointegration process [29].

The internal portion of the bone, in the present study, does not indicate any statistical difference between the different groups regarding the IID nor the type of surgical preparation protocol. This underscores the resilience of the biological healing process in the face of mechanical forces. One explanation could be related to the macrogeometry of the implant used in the present study.

Hansson [30] also emphasized the significance of macro- and micro-designs of implants in distributing forces around the peri-implant bone. It was shown that implants featuring micro-threads are biomechanically beneficial. The microthreads helps in reducing peak stresses at the interface with the marginal bone, aiding in the bone modelling and remodelling processes [31].

One limitation of the present study is the use of an animal model which do not fully replicate human oral anatomy and bone healing processes. The short observation period (5 weeks**)** captures early healing events and may not reflect long-term outcomes and the complete remodelling and maturation of bone around the implants. A second limitation is the evaluation of only a single undersizing dimension (0.3 mm) and remains unknown whether a smaller undersize (e.g. 0.1–0.2 mm) would produce a milder response, or a larger undersize (e.g. 0.5 mm) would induce even more microdamage and remodeling. However, our methodology was in line with prior in vivo publications [15, 21].

This limitation highlights the need for clinical trials to validate the findings in human subjects to better understand the complex interplay between mechanical forces and biological healing in dental implantology.

## Conclusions

The study found no statistically significant differences between osteotomy preparation protocols (undersized vs. non-undersized) or inter-implant distances (2 mm vs. 4 mm) for the evaluated parameters. However, a trend of increased bone remodelling and higher bone-to-implant contact (BIC) was observed at 2 mm IID, particularly with the undersized protocol. These findings highlight the complexity of the interplay between mechanical forces and biological healing, warranting further clinical studies to validate these observations. Clinicians should exercise caution during immediate loading, especially when inserting two implants close together with high torque.

## Supplementary Information

Supplementary Material 1.

## Data Availability

No datasets were generated or analysed during the current study.

## Abbreviations

BAFO: Bone area fraction occupancy
BIC: Bone-to-implant contact
BMD: Bone mineral density
BVTV: Bone volume fraction
IID: Inter-implant distance
ITQ: Insertion torque
NUP: Non-undersized preparation
ROI: Region of interest
UP: Undersized preparation
VOI: Volume of interest

## References

[1] Scarano A, Assenza B, Piattelli M, Thams U, Roman FS, Favero GA, Piattelli A. Interimplant distance and crestal bone resorption: a histologic study in the canine mandible. Clin Implant Dent Relat Res. 2004;6(3):150–6. 15726849 10.1111/j.1708-8208.2004.tb00222.x

[2] Teughels W, Merheb J, Quirynen M. Critical horizontal dimensions of interproximal and buccal bone around implants for optimal aesthetic outcomes: a systematic review. Clin Oral Implants Res. 2009;20(Suppl 4):134–45. 19663960 10.1111/j.1600-0501.2009.01782.x

[3] Koutouzis T, Neiva R, Upton D, Lundgren T. The effect of interimplant distance on Peri-implant bone and soft tissue dimensional changes: A Nonrandomized, Prospective, 2-Year Follow-up study. Int J Oral Maxillofacial Implants. 2015;30(4):900‑8. 10.11607/jomi.4040 26252042

[4] Tarnow D, Cho S, Wallace S. The effect of inter-implant distance on the height of inter-implant bone crest. J Periodontol. 2000;71(4):546–9. 10807116 10.1902/jop.2000.71.4.546

[5] Cardaropoli G, Wennström JL, Lekholm U. Peri-implant bone alterations in relation to inter-unit distances: a 3-year retrospective study. Clin Oral Implants Res. 2003;14(4):430–6. 12869005 10.1034/j.1600-0501.2003.00895.x

[6] Chang M, Wennstrom JL. Bone alterations at implant-supported FDPs in relation to inter-unit distances: a 5-year radiographic study. Clin Oral Implants Res. 2010;21(7):735–40. 20384704 10.1111/j.1600-0501.2009.01893.x

[7] Kourkouta S, Dedi KD, Paquette DW, Mol A. Interproximal tissue dimensions in relation to adjacent implants in the anterior maxilla: clinical observations and patient aesthetic evaluation. Clin Oral Implants Res. 2009;20(12):1375–85. 19681967 10.1111/j.1600-0501.2009.01761.x

[8] Danza M, Zollino I, Avantaggiato A, Lucchese A, Carinci F. Distance between implants has a potential impact of crestal bone resorption. Saudi Dent J. 2011;23(3):129–33. 23960506 10.1016/j.sdentj.2011.02.002 PMC3723294

[9] Friberg B, Sennerby L, Gröndahl K, Bergström C, Bäck T, Lekholm U. On cutting torque measurements during implant placement: a 3-year clinical prospective study. Clin Implant Dent Relat Res. 1999;1(2):75–83. 11359301 10.1111/j.1708-8208.1999.tb00095.x

[10] Natali AN, Carniel EL, Pavan PG. Investigation of viscoelastoplastic response of bone tissue in oral implants press fit process. J Biomed Mater Res B Appl Biomater. 2009;91(2):868–75. 19637368 10.1002/jbm.b.31469

[11] O’Mahony AM, Williams JL, Katz JO, Spencer P. Anisotropic elastic properties of cancellous bone from a human edentulous mandible. Clin Oral Implants Res. 2000;11(5):415–21. 11168233 10.1034/j.1600-0501.2000.011005415.x

[12] Stocchero M, Jinno Y, Toia M, Ahmad M, Papia E, Yamaguchi S, et al. Intraosseous temperature change during installation of dental implants with two different surfaces and different drilling protocols: an in vivo study in sheep. J Clin Med. 2019;8(8):1198. 31405207 10.3390/jcm8081198 PMC6723378

[13] Halldin A, Jimbo R, Johansson CB, Wennerberg A, Jacobsson M, Albrektsson T, Hansson S. The effect of static bone strain on implant stability and bone remodeling. Bone. 2011;49(4):783–9. 21782050 10.1016/j.bone.2011.07.003

[14] Wang L, Ye T, Deng L, Shao J, Qi J, Zhou Q, et al. Repair of microdamage in osteonal cortical bone adjacent to bone screw. PLoS ONE. 2014;9(2):e89343. 24586702 10.1371/journal.pone.0089343 PMC3930719

[15] Stocchero M, Toia M, Jinno Y, Cecchinato F, Becktor JP, Naito Y, et al. Influence of different drilling preparation on cortical bone: a biomechanical, histological, and micro-CT study on sheep. Clin Oral Implants Res. 2018;29(7):707–15. 29781224 10.1111/clr.13262

[16] Stocchero M, Jinno Y, Toia M, Jimbo R, Lee C, Yamaguchi S, et al. In silico multi-scale analysis of remodeling peri-implant cortical bone: a comparison of two types of bone structures following an undersized and non-undersized technique. J Mech Behav Biomed Mater. 2020;103:103598. 32090927 10.1016/j.jmbbm.2019.103598

[17] Degidi M, Novaes AB Jr, Nardi D, Piattelli A. Outcome analysis of immediately placed, immediately restored implants in the esthetic area: the clinical relevance of different interimplant distances. J Periodontol. 2008;79(6):1056–61. 18533783 10.1902/jop.2008.070534

[18] McNeish D, Stapleton LM. Modeling clustered data with very few clusters. Multivar Behav Res. 2016;51(4):495–518. 10.1080/00273171.2016.1167008 27269278

[19] De Angelis P, Liguori MG, Rella E, Staffieri A, Piccirillo D, Manicone PF. Influence of the interimplant horizontal distance on the clinical outcomes of patients rehabilitated with two adjacent implants: a systematic review and meta-analysis. J Prosthet Dent. 2025. 10.1016/j.prosdent.2025.06.004. 40628575 10.1016/j.prosdent.2025.06.004

[20] Simsek B, Erkmen E, Yilmaz D, Eser A. Effects of different inter-implant distances on the stress distribution around endosseous implants in posterior mandible: a 3D finite element analysis. Med Eng Phys. 2006;28(3):199–213. 15979921 10.1016/j.medengphy.2005.04.025

[21] Stocchero M, Jinno Y, Toia M, Ahmad M, Galli S, Papia E, et al. Effect of drilling preparation on immediately loaded implants: an in vivo study in sheep. Int J Oral Maxillofac Implants. 2023. 10.11607/jomi.9949. 37279224 10.11607/jomi.9949

[22] Duyck J, Corpas L, Vermeiren S, Ogawa T, Quirynen M, Vandamme K, et al. Histological, histomorphometrical, and radiological evaluation of an experimental implant design with a high insertion torque. Clin Oral Implants Res. 2010;21(8):877–84. 20528892 10.1111/j.1600-0501.2010.01895.x

[23] Halldin A, Jimbo R, Johansson CB, Wennerberg A, Jacobsson M, Albrektsson T, et al. Implant stability and bone remodeling after 3 and 13 days of implantation with an initial static strain. Clin Implant Dent Relat Res. 2014;16(3):383–93. 23061968 10.1111/cid.12000

[24] Taguchi T, Lopez MJ. An overview of *de Novo* bone generation in animal models. J Orthop Res. 2021;39(1):7–21. 32910496 10.1002/jor.24852 PMC7820991

[25] Rosas-Díaz J, Guerrero ME, Córdova-Limaylla N, Galindo-Gómez M, García-Luna M, Cayo-Rojas C. The influence of the degree of dental implant insertion compression on primary stability measured by resonance frequency and progressive insertion torque: in vitro study. Biomedicines. 2024. 10.3390/biomedicines12122878. 39767784 10.3390/biomedicines12122878 PMC11672996

[26] Slaets E, Carmeliet G, Naert I, Duyck J. Early cellular responses in cortical bone healing around unloaded titanium implants: an animal study. J Periodontol. 2006;77(6):1015–24. 16734577 10.1902/jop.2006.050196

[27] Tomasi C, Sanz M, Cecchinato D, Pjetursson B, Ferrus J, Lang NP, et al. Bone dimensional variations at implants placed in fresh extraction sockets: a multilevel multivariate analysis. Clin Oral Implants Res. 2010;21(1):30–6. 20070744 10.1111/j.1600-0501.2009.01848.x

[28] Araújo MG, Silva CO, Souza AB, Sukekava F. Socket healing with and without immediate implant placement. Periodontol 2000. 2019;79(1):168–77. 30892762 10.1111/prd.12252

[29] Natali AN, Carniel EL, Pavan PG. Dental implants press fit phenomena: biomechanical analysis considering bone inelastic response. Dent Mater. 2009;25(5):573–81. 19128827 10.1016/j.dental.2008.11.002

[30] Hansson S. The implant neck: smooth or provided with retention elements. A biomechanical approach. Clin Oral Implants Res. 1999;10(5):394–405. 10551064 10.1034/j.1600-0501.1999.100506.x

[31] Hansson S. A conical implant–abutment interface at the level of the marginal bone improves the distribution of stresses in the supporting bone: an axisymmetric finite element analysis. Clin Oral Implants Res. 2003;14(3):286–93. 12830797 10.1034/j.1600-0501.2003.140306.x

